# Continuity of Care in Persistent Delusional Disorder: The Role of the Family Doctor

**DOI:** 10.7759/cureus.110193

**Published:** 2026-06-03

**Authors:** Diogo C Martins, Nuno M Nunes, Sara V Garrido, Inês R Neves, Margarida G Silva

**Affiliations:** 1 Family Medicine, Unidade de Saúde Familiar (USF) Gago Coutinho, Unidade Local de Saúde (ULS) do Estuário do Tejo, Alverca do Ribatejo, PRT

**Keywords:** collaborative care, continuity of patient care, delusional disorder, family medicine, involuntary hospitalization, patient trust, primary care medicine, severe mental illness, therapeutic alliance, therapeutic engagement

## Abstract

Family doctors (FDs) play a central role in the early recognition and longitudinal management of severe mental illness (SMI), often acting as gatekeepers to psychiatric care. However, some patients refuse mental health services due to limited insight, stigma, or mistrust. In such cases, continuity of care and a strong therapeutic relationship in primary care can be crucial in facilitating engagement.

We report the case of a 62-year-old man who presented to the emergency department after ingesting multiple paracetamol tablets for abdominal pain. In light of the persecutory delusions observed during assessment, psychiatric evaluation was requested, and hospitalization was recommended. The patient, however, refused admission and did not attend follow-up. During subsequent primary care visits, he remained delusional and declined psychiatric referral. Over the following year, his FD maintained regular follow-up, and a consistent empathetic approach gradually earned the patient’s trust. This was followed by the patient’s acceptance of referral and attendance at an initial psychiatric appointment, although he subsequently disengaged from follow-up once again. Due to clinical deterioration, involuntary psychiatric admission was required. He was diagnosed with persistent delusional disorder, and after 23 days of antipsychotic treatment, his condition improved significantly. Following discharge, he remained clinically stable with ongoing psychiatric follow-up.

This case highlights the role of FDs in maintaining patient engagement when psychiatric care is initially refused. FD continuity of care may help facilitate later engagement with psychiatric services and improve access to treatment in patients with SMI.

## Introduction

Severe mental illness (SMI) encompasses a group of chronic psychiatric conditions (such as schizophrenia spectrum disorders and persistent delusional disorder) that significantly affect social functioning, quality of life, and physical health [1,2]. Individuals with SMI experience higher rates of cardiovascular disease, substance use, and premature mortality compared with the general population, reinforcing the need for integrated and longitudinal care [2].

Patients with SMI often interact with the healthcare system through multiple entry points, but initial symptoms frequently emerge in primary care settings. Family doctors (FDs) are commonly the first clinicians to identify changes in behaviour, mood, or daily functioning and to initiate referral to psychiatric services [3,4]. This frontline role is well established in primary care literature, where general practitioners are recognized as key providers in the early detection and ongoing management of mental health disorders [3,5].

However, the pathway from first presentation to adequate psychiatric follow-up is often complex. Stigma, fear, impaired insight, and mistrust of mental health services may delay diagnosis and treatment [6]. In such cases, FDs are responsible not only for monitoring physical comorbidities and risk factors but also for maintaining engagement with patients who may refuse both pharmacological treatment and psychiatric evaluation. This longitudinal relationship is a defining feature of family medicine and has been associated with improved adherence, greater patient satisfaction, and better clinical outcomes [7,8].

Over time, continuity of care may allow trust to develop in ways that a single psychiatric encounter cannot achieve. The present case illustrates how sustained engagement in primary care can help overcome initial resistance to treatment and facilitate access to specialized services. It highlights the practical and often underrecognized role of FDs in the early and intermediate phases of SMI, where therapeutic alliance may be as important as pharmacological intervention in determining outcomes.

A preliminary version of this work was previously presented as a poster at the WONCA World 2025 Conference, held in Lisbon, Portugal, from September 17 to 21, 2025, under the title “The Family Doctor’s Role in Severe Mental Illness: Building Trust for Better Outcomes.”

## Case presentation

**Figure 1:**
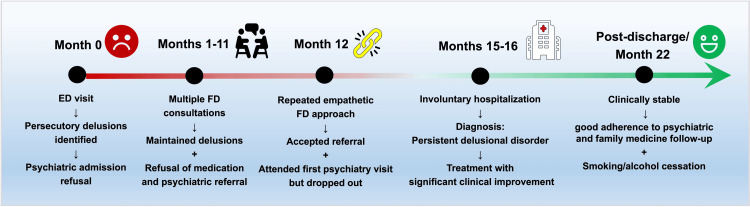
Clinical timeline of patient management from initial presentation to post-discharge stabilization. Calendar dates were masked for confidentiality. Month 0 refers to the initial emergency department presentation. The timeline summarizes the patient’s progression from identification of persecutory delusions and refusal of psychiatric admission, through repeated family doctor follow-up and eventual psychiatric referral, to involuntary hospitalization, treatment initiation, and post-discharge clinical stability. ED: Emergency Department; FD: Family Doctor. This figure was created using Microsoft PowerPoint (Microsoft Corporation, Redmond, WA, US).

The patient was a 62-year-old man with a medical history of hypertension, arrhythmia, chronic obstructive pulmonary disease (COPD), active smoking of 15 cigarettes per day, and regular alcohol consumption of approximately 1 L of beer per day. For confidentiality, calendar dates are reported as relative time points, with Month 0 referring to the initial emergency department presentation. At Month 0, the patient presented to the emergency department after reportedly ingesting five 1-g paracetamol tablets and an unspecified quantity of ibuprofen for abdominal pain that he attributed to being poisoned by family members. Initial medical assessment, including blood tests, serum paracetamol/acetaminophen measurement, and blood alcohol level, showed no acute organ damage, clinically significant paracetamol toxicity, or alcohol intoxication. A psychiatric evaluation was requested to assess suicidal intent and suspected delusional thinking. During the assessment, he denied suicidal intent and exhibited structured persecutory delusional beliefs, asserting that family members were attempting to poison him and were involved in organized crime. Psychiatric hospitalization was recommended; however, the patient refused admission. As he did not meet the legal criteria for compulsory hospitalization at that time, he was discharged with olanzapine 5 mg and advised to attend outpatient psychiatric follow-up, which he did not pursue. The chronological progression of the case is summarized in Figure 1.

One month later, he was first evaluated by his FD. At that time, he continued to report persistent persecutory delusions. He had not initiated the prescribed olanzapine, reporting delusional mistrust toward psychiatric treatment and stating that he believed the psychiatrist intended to harm him. He again declined psychiatric referral. Over the following months, the FD maintained regular follow-up appointments, addressing the patient’s physical comorbidities while gradually revisiting the possibility of psychiatric evaluation.

After approximately 11 months, following repeated discussions and an empathetic therapeutic approach, the patient agreed to attend a psychiatric consultation. Until that point, he had repeatedly refused psychiatric referral because he lacked insight and held delusional beliefs about psychiatric treatment, including the belief that the psychiatrist intended to harm him. The FD’s approach relied on regular, non-confrontational consultations, continued management of physical comorbidities, and gradual reintroduction of the need for psychiatric evaluation. This sustained engagement appeared to reduce resistance sufficiently for the patient to accept an initial psychiatric appointment. Although he attended the first consultation at Month 12, he subsequently disengaged from follow-up and remained non-adherent to treatment.

Over the following three months, his symptoms persisted and were associated with increasing functional impairment. He remained convinced that family members were attempting to poison or harm him, lacked insight into the pathological nature of these beliefs, and became increasingly socially withdrawn. The FD maintained regular follow-up and, after noting clinical worsening and persistent refusal of voluntary care, communicated with the psychiatry service.

At Month 15, following this communication, the psychiatry service requested a formal transport warrant from the local public health authority for urgent psychiatric assessment and possible hospitalization. The warrant was issued in view of the patient’s longstanding psychotic condition, systematized persecutory delusions with marked affective-behavioral impact, refusal of treatment, and refusal of voluntary clinical follow-up. He was subsequently brought to the emergency department, where a psychiatric assessment documented persistent persecutory and poisoning delusions, including the belief that family members were trying to kill him. He lacked insight and refused hospitalization. Mental status examination revealed limited cooperation, pressured and difficult-to-interrupt speech, dysphoric mood, delusions of harm and poisoning, and no suicidal or homicidal ideation. Hospitalization was proposed for clinical stabilization, but the patient refused admission. Involuntary psychiatric admission was therefore carried out under the Portuguese Mental Health Law.

During hospitalization, a comprehensive psychiatric assessment confirmed the diagnosis of persistent delusional disorder. Antipsychotic treatment with a long-acting injectable formulation of paliperidone was initiated. The patient tolerated treatment well and demonstrated progressive clinical improvement, including reduced anxiety, diminished behavioral impact of his delusional beliefs, improved self-care, and partial distancing from the previously dominant persecutory themes.

After 23 days of inpatient treatment, he was discharged with outpatient psychiatric follow-up and continued long-acting paliperidone therapy. At subsequent follow-up visits, he remained clinically stable, adhered to his psychiatric treatment regimen, and maintained regular appointments. Concurrently, he demonstrated improved engagement with primary care, including management of COPD and cardiovascular risk factors. At Month 22, he reported cessation of both tobacco and alcohol use and maintained stable physical and mental health under coordinated follow-up between psychiatry and family medicine.

## Discussion

This case illustrates several challenges commonly encountered in the management of SMI in community settings. Patients with psychotic disorders frequently present outside specialized psychiatric services, often through primary care or emergency departments. In such contexts, the FD assumes a central role, not only in recognizing emerging psychiatric symptoms but also in managing physical comorbidities and navigating the patient’s attitudes toward mental health care [3,4].

A key challenge relates to insight and adherence. Limited insight is a well-recognized feature of psychotic disorders and may significantly impair treatment adherence and engagement with mental health services [9]. In our patient, the absence of suicidal intent and relatively preserved functioning initially made involuntary admission neither clinically nor legally justified. As a result, the FD remained the main point of contact for nearly a year, despite persistent delusional beliefs. During this period, continuity of care became clinically meaningful. Regular consultations allowed not only monitoring of cardiovascular and respiratory disease but also the maintenance of a therapeutic relationship that the patient trusted.

**Figure 2:**
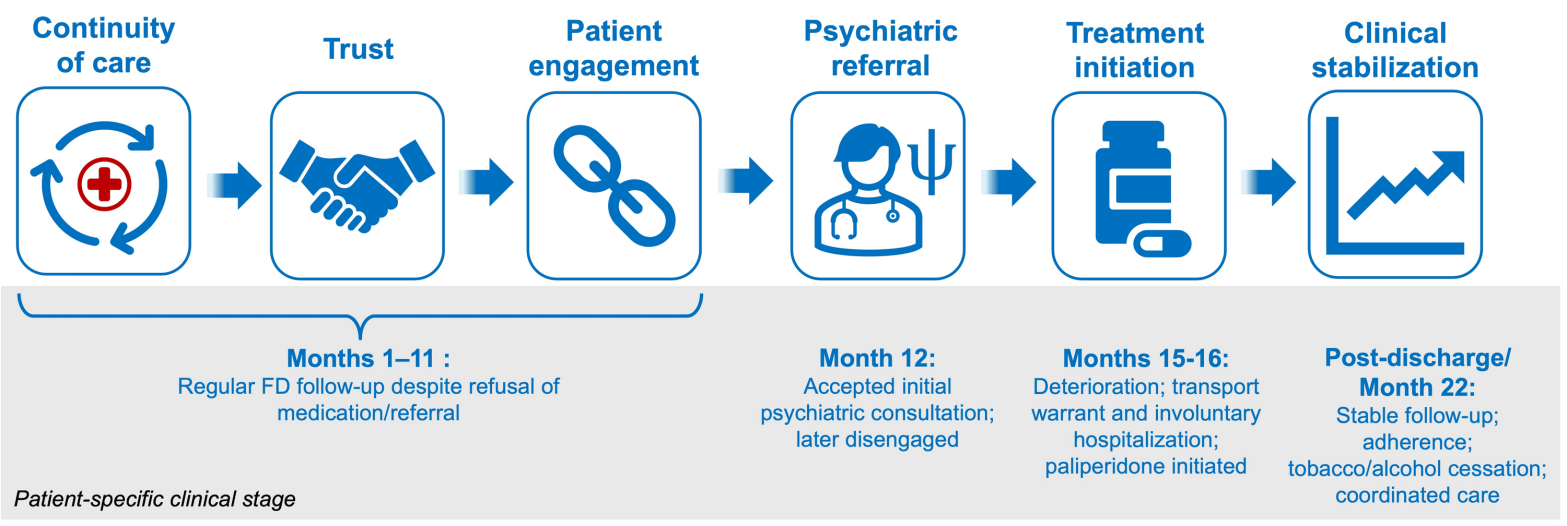
Case-applied conceptual model of the family doctor’s role in facilitating engagement and stabilization in severe mental illness. The figure links core primary care functions to the patient’s clinical course. Initial FD contact allowed continued follow-up after emergency department discharge and refusal of psychiatric care. Over the following months, continuity and trust-building through regular FD consultations created repeated opportunities to revisit psychiatric referral. The patient later accepted an initial psychiatric consultation but disengaged again, requiring escalation after clinical deterioration. Involuntary hospitalization enabled treatment initiation, and subsequent coordinated follow-up between psychiatry and family medicine supported clinical stabilization, medication adherence, and management of physical comorbidities. FD: family doctor This figure was created using Microsoft PowerPoint (Microsoft Corporation, Redmond, WA, US).

In the present case, trust appeared to precede adherence. Continuity of care and a sustained therapeutic relationship in primary care fostered patient trust and created repeated opportunities to revisit psychiatric referral in a non-confrontational manner, eventually facilitating the patient’s initial engagement with psychiatric services. This progression from refusal of care to treatment engagement and stabilization is summarized in a case-applied conceptual model (Figure 2). The observed course is consistent with evidence that therapeutic alliance is associated with improved medication adherence and engagement with mental health services [10,11], and with humanized and patient-centered care models, in which the quality of the doctor-patient relationship is considered a key determinant of engagement and clinical outcomes [8,12]. Longitudinal primary care relationships may also support motivational dialogue, reduce stigma-related avoidance, facilitate early identification of clinical deterioration, and improve coordination across levels of care [3,7]. The value of regular medical contact is further supported by population-based evidence suggesting that regular visits to medical institutions, including primary care, may mitigate suicide risk during periods of increased psychosocial vulnerability [13].

The FD’s contribution in this case can be further understood through the ACCCA framework of primary care: Accessibility, Comprehensiveness, Coordination, Continuity, and Accountability [14]. Accessibility was reflected in the patient’s repeated contact with primary care despite refusing psychiatric services. Comprehensiveness was demonstrated by the simultaneous management of psychiatric symptoms, cardiovascular risk factors, COPD, tobacco use, and alcohol consumption. Coordination was central to facilitating psychiatric referral and later supporting follow-up after hospitalization. Continuity allowed the therapeutic relationship to develop over time, creating repeated opportunities to revisit psychiatric care without confrontation. Finally, accountability was reflected in the FD’s ongoing responsibility for risk monitoring, follow-up, and prevention of care fragmentation. This framework helps contextualize the broader contribution of primary care beyond continuity alone.

Recent literature also supports discussing this case within the broader context of collaborative and integrated care. Collaborative mental health care generally refers to structured integration between primary care, care managers, and psychiatric consultants, aiming to improve access to mental health treatment and coordination across services [15]. For people with SMI specifically, collaborative care approaches have been developed to improve interdisciplinary working between primary and secondary care, although an updated Cochrane review found that available evidence does not clearly demonstrate superiority over standard or non-collaborative care in medium-term outcomes [16]. Nevertheless, primary care-based models such as PARTNERS2 illustrate the continued relevance of shared-care approaches for people with schizophrenia, bipolar disorder, and other psychoses living in the community [17]. In the present case, this is reflected in the FD’s role in maintaining contact, monitoring deterioration, and coordinating escalation to psychiatric services when voluntary engagement failed.

As often observed in clinical practice, initial engagement does not necessarily translate into sustained adherence. In this case, the patient disengaged from psychiatric follow-up after the initial consultation, with subsequent clinical deterioration. The FD’s ongoing follow-up was important in recognizing this deterioration and communicating with psychiatric services when voluntary engagement was no longer effective. This collaboration contributed to urgent psychiatric reassessment and, ultimately, involuntary hospitalization for stabilization under the appropriate legal framework. When psychiatric risk persists and voluntary treatment fails, involuntary hospitalization may become necessary to prevent further deterioration or potential harm [18]. Although it should remain a last resort, involuntary admission in this case allowed formal diagnosis, initiation of antipsychotic treatment, and subsequent clinical stabilization.

Beyond psychiatric stabilization, this case also highlights the importance of integrated care. Individuals with SMI have disproportionately higher rates of cardiovascular disease and reduced engagement in preventive health measures [2,19]. Primary care teams are uniquely positioned to monitor and address these risks through a holistic approach. In the Portuguese context, concerns regarding insufficient monitoring of cardiovascular risk factors, vaccination, and preventive screening in individuals with SMI have also been raised in the medical literature, reinforcing the importance of coordinated care between primary and secondary services [20]. In our patient, improved psychiatric stability was accompanied by better engagement with primary care, including management of COPD and cardiovascular risk factors, as well as cessation of alcohol and tobacco use.

This case underscores the often underrecognized role of FDs in the management of SMI. In addition to addressing physical health needs, which are frequently neglected in this population, FDs act as intermediaries between patients and psychiatric services. In settings where insight is limited and stigma remains a barrier, the longitudinal nature of primary care allows trust to develop over time, which may ultimately determine access to appropriate treatment and long-term outcomes.

## Conclusions

This case highlights the critical role of FDs in the management of SMI, particularly in patients with limited insight and initial resistance to care. In such situations, primary care may become the only consistent point of contact, allowing for early recognition of psychiatric symptoms, ongoing monitoring, and gradual facilitation of specialist referral. In our patient, continuity of care and a sustained therapeutic relationship enabled eventual engagement with psychiatric services, while also supporting the management of physical comorbidities.

Although involuntary hospitalization ultimately marked the turning point in clinical stabilization, the groundwork established in primary care through trust, persistence, and a holistic approach appeared to contribute to sustained improvement. This case supports the role of primary care within mental health systems, particularly in helping to bridge gaps between hospital and community care, reduce care fragmentation, and support ongoing recovery in patients with SMI.
